# Agroforestry coffee soils increase the insect‐suppressive potential offered by entomopathogenic fungi over full‐sun soils: A case proposing a “bait survival technique”

**DOI:** 10.1002/ece3.5598

**Published:** 2019-08-30

**Authors:** Camila Costa Moreira, Daiane Celestino, Tathiana Guerra Sobrinho, Irene Maria Cardoso, Simon Luke Elliot

**Affiliations:** ^1^ Departamento de Entomologia Universidade Federal de Viçosa Viçosa MG Brazil; ^2^ Departamento de Biologia Geral, Ecologia Universidade Federal de Viçosa Viçosa MG Brazil; ^3^ Departamento de Ciências Agrárias e Biológicas Universidade Federal do Espírito Santo São Mateus ES Brazil; ^4^ Departamento de Solos Universidade Federal de Viçosa Viçosa MG Brazil

**Keywords:** bait survival technique, ecosystem services, entomopathogenic fungi, insect bait method, speed of kill, suppressive soil, survival analysis

## Abstract

Entomopathogenic fungi are important natural enemies of insects. However, there is little information on the insect‐suppressive potential of these fungi and possible effects of farming management on this. Meanwhile, changes in natural landscapes due to agricultural intensification have caused considerable biodiversity loss and consequent decay of ecosystem services. However, the adoption of practices such as agroforestry in agroecosystems can foster abiotic and biotic conditions that conserve biodiversity, consequently restoring the provision of ecosystems services. Here, we assessed the effect of management systems (agroforestry or full‐sun) on the pest‐suppressive potential of entomopathogenic fungi in Brazilian coffee plantations. We used the insect bait method coupled with survival analyses to assess the speed of kill by entomopathogenic fungi and their presence in soil samples from both farming systems. We found that insects exposed to agroforestry soils died more quickly than insects exposed to full‐sun soils. Of the fungi isolated from the bait insects, *Metarhizium* was found most frequently, followed by *Beauveria*. Meanwhile, *Fusarium* was frequently isolated as primary or secondary infections. We propose that the differential survival of insects is indicative of a greater suppressive potential by entomopathogenic fungi in agroforestry, and that this could be promoted by the diversified landscape, microclimatic stability, and reduced soil disturbance in agroforestry systems. Furthermore, our results provide a useful demonstration of the potential use of the insect bait method to investigate pest‐suppressive potential through bait insect mortality, and we term this the “bait survival technique.”

## Graphical abstract

Coffee agroforestry soils increase “soil insect suppressiveness” by entomopathogenic fungi in relation to full‐sun coffee soils. The insect suppressive potential was assessed by an adaptation of the insect bait method coupled with survival analysis. This enabled us to take the first steps to evaluate an important ecosystem service provided by entomopathogenic fungi.

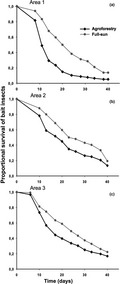

## 1 INTRODUCTION

Changes in natural landscapes due to agricultural intensification have caused considerable biodiversity loss and consequent decay of ecosystem services (Matson, Parton, Power, & Swift, 1997). This intensification includes the dedication of extensive areas to monoculture, resulting in simplified agroecosystems. Thus, 60 years after the Green Revolution, which propelled agricultural intensification, it has become evident that there is a need for more suitable forms of agricultural production, including the adoption of agroecological systems and technologies that emphasize conservation and regeneration of ecosystem services (Pingali, 2012; Tilman, 1998). Agroecological systems such as agroforestry can mimic forest natural habitats (Altieri, 1999; Lin, 2007), promoting shaded soil, reducing microclimatic variation, conserving moisture, and reducing ecosystem disturbance (Jose, 2009), thereby potentially improving the maintenance of ecosystem services and soil quality, especially for tropical understory plants such as cacao and coffee (Cardoso, Boddington, Janssen, Oenema, & Kuyper, 2003; De Beenhouwer, Aerts, & Honnay, 2013; Tscharntke et al., 2011).

Coffee plants are originally forest understory shrubs and as crops are traditionally grown under a canopy of shade trees (Staver, Guharay, Monterroso, & Muschler, 2001). The agroforestry system together with the perennial nature of coffee cultivation creates a stable environment that can represent a refugium for a plethora of organisms, particularly in deforested areas (Perfecto, Rice, Greenberg, & Voort, 1996). Many of these organisms are natural enemies of insect pests including birds, bats and predatory or parasitoid arthropods (Klein, Steffan‐Dewenter, & Tscharntke, 2006; Letourneau, Jedlicka, Bothwell, & Moreno, 2009; Philpott & Armbrecht, 2006; Rezende, Venzon, Perez, Cardoso, & Janssen, 2014; Tylianakis, Klein, & Tscharntke, 2005; Tylianakis, Tscharntke, & Klein, 2006).

Pest control by pre‐existing natural enemies (or conservation biological control) is a major ecosystem service that is promoted by biodiversity (Iverson et al., 2014; Pell, Hannam, & Steinkraus, 2010; Tscharntke et al.., 2015; Wilby & Thomas, 2002), yet paradoxically pest control is one of the main factors driving the use of agrochemicals in conventional agriculture and resultant loss of biodiversity. Of the naturally occurring enemies that can provide natural pest control, insect‐pathogenic microorganisms are perhaps the most neglected, with most of the studies focusing in entomophagous natural enemies such as birds, insects predators, and parasitoids (Bengtsson, Ahnström, & Weibull, 2005; Chaplin‐Kramer, O'Rourke, Blitzer, & Kremen, 2011; Hatt, Boeraeve, Artru, Dufrêne, & Francis, 2018; Letourneau et al., 2011). To date, the role of entomopathogenic fungi–especially those within the Hypocreales–as providers of ecosystem services in a natural context has received little attention, despite the cosmopolitan distribution of these natural enemies, their abundance and their potential to impact insect populations in soils of natural and cultivated areas (Hesketh, Roy, Eilenberg, Pell, & Hails, 2010). The hypocrealean fungi *Metarhizium* and *Beauveria* have been subjected to intense study over the last 200 years, largely aimed at their use as bioinsecticides in inundative biological control programs (Vega et al., 2009). More recently, the importance of their ecology, occurrence and abundance (Kepler, Ugine, Maul, Cavigelli, & Rehner, 2015; Meyling & Eilenberg, 2006, 2007; Sharma, Oliveira, Torres, & Marques, 2018), and as plant mutualists has come to the fore (Barelli, Moreira, & Bidochka, 2018; Behie et al., 2017; Behie, Zelisko, & Bidochka, 2012; Bruck, 2010; Elliot et al., 2000).

Meanwhile, in the field of plant pathology, the concept of plant disease suppressive soils as a form of (conservation) biological control is well established and is considered as an ecosystem service (Bailey & Lazarovits, 2003; Brussaard, Ruiter, & Brown, 2007; Mazzola, 2004). In such suppressive soils, plant pathogens are less likely to establish or persist, and when they are able to establish, they cause little damage (Hornby, 1983). The natural control that entomopathogenic fungi exert on soil insects can be considered a parallel to this and thus be considered an ecosystem service.

The cryptic nature of entomopathogenic fungi in soils hinders assessment of their provision of ecosystem services (suppression of soil‐dwelling pest insects). However, many studies have assessed the occurrence and abundance of these fungi in natural and cultivated soils across the world (Ali‐Shtayeh, Mara'i, & Jamous, 2003; Bidochka, Kasperski, & Wild, 1998; Clifton, Jaronski, Hodgson, & Gassmann, 2015; Goble, Dames, Hill, & Moore, 2010, Klingen, Eilenberg, & Meadow, 2002; Meyling & Eilenberg, 2006; Quesada‐Moraga, Navas‐Cortés, Maranhao, Ortiz‐Urquiza, & Santiago‐Álvarez, 2007; Vanninen, 1996). Some of these studies, comparing the occurrence of entomopathogenic fungi in different agricultural management systems, report an increase in the occurrence of entomopathogenic fungi in organic or otherwise more sustainably managed systems (Clifton et al., 2015; Goble et al., 2010; Jabbour & Barbercheck, 2009; Klingen et al., 2002). These studies have contributed to our understanding of the diversity, distribution, and abundance of some genera of hypocrealean entomopathogenic fungi, in addition to raising new questions about their ecology and the associations with other organisms in which they engage. However, open questions are how the presence of these organisms may translate into the many ecological functions that can be performed by them as insect pathogens or plant mutualists. More specifically, here we are interested in how their presence may translate to the control of insect populations. Information of this nature could ultimately lead to practical suggestions regarding conservative biological control of pest insects, rather than being restricted to lists of species and their abundances.

In this study, we aimed to compare potential ecosystem services provided by entomopathogenic fungi in two coffee management systems: Agroforestry and full‐sun. Studies of coffee agroforestry systems have shown positive effects of this system on pest control by entomophagous natural enemies (Karp et al., 2013; Rezende et al., 2014) and also on soil organisms (Cardoso et al., 2003; Velmourougane, 2017). Based on this, we hypothesize that (a) entomopathogenic fungi in coffee agroforestry will show a greater speed of kill of bait insects and (b) be more abundant than the fungi from full‐sun managed soil. For this, we used an adaptation of insect bait method coupled with survival analyses of the bait insects, referred here as “bait survival technique,” and also accounted for the occurrence of fungal genera recovered with baits.

## 2 MATERIALS AND METHODS

### 2.1 Study area

**Figure 1:**
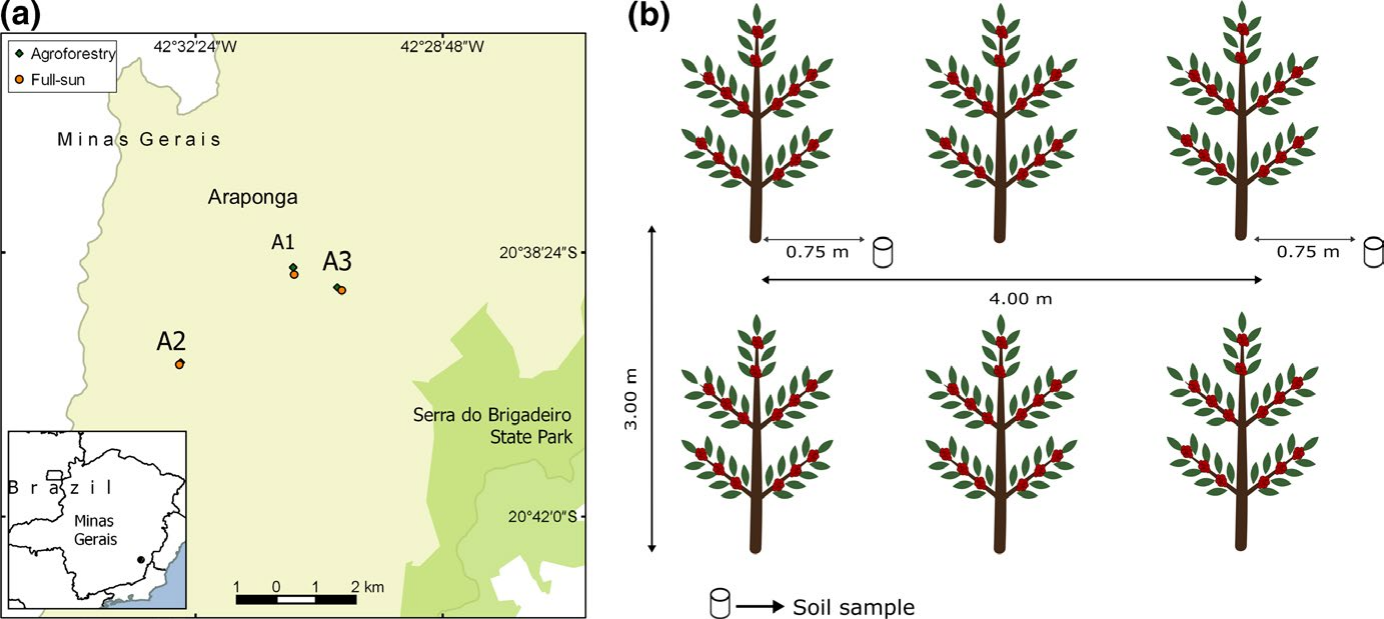
Geographical location of sample areas in Araponga, Minas Gerais, southeastern Brazil and the sample scheme. (a) Sample areas Area 1 (A1), Area 2 (A2), and Area 3 (A3), each containing two fields: Agroforestry and full‐sun. (b) Sample scheme showing the distances between each sample and between coffee rows

The study was conducted in the municipality of Araponga (Figure 1a), Minas Gerais, southeastern Brazil (20°48′S and 42 32′W). This municipality is in the Zona da Mata region, within the Atlantic Coastal Rainforest biome, a biodiversity hotspot. It is characterized by a tropical highland climate with mean temperature and precipitation of 18°C and 1,500 mm. Coffee is the cash crop in the region, and the dominant soil is Oxisoil, this being acidic and poor in available nutrients (Cardoso, Guijt, Franco, Carvalho, & Neto, 2001; Mendonça and Stott, 2003). Sampling was conducted on smallholdings under coffee (*Coffea arabica* L.) cultivation. The sampled areas are located in the vicinity of the Serra do Brigadeiro State Park (Figure 1a), a remnant of Atlantic Forest in the mountainous region of Minas Gerais. Soil samples were taken in three areas containing two coffee fields each under a different management system: organic agroforestry systems or full‐sun systems (i.e., paired samples in six fields, three agroforestry and three full‐sun; Figure 1a; Table 1). Both fields in each area presented very similar characteristics regarding the geographical location, age of coffee plants and are managed by the same farmer or the same family of farmers. The agroforestry fields had native and non‐native shade trees planted between coffee rows, fertilization was provided by green and animal manure, and spontaneous noncrop plants were left in the rows. The full‐sun fields are characterized by unshaded open cultivation with addition of inorganic fertilizers and weeding of spontaneous plants. No pesticides or biological methods of pest control were used in either management system in the year of sampling.

**Table 1:** Soil variables and geographical characteristics of the fields in each sample field

Soil factors	Agroforestry			Full‐sun		
	A1	A2	A3	A1	A2	A3
pH (in H_2_O)	6.18	6.03	6.02	5.97	5.36	5.60
MO (%)	5.12	4.61	5.72	4.09	2.94	4.92
Sand (%)	41	44	39	33	39	49
Silt (%)	7	8	15	8	14	12
Clay (%)	52	48	46	52	47	39
Classification	Clay	Clay	Clay	Clay	Clay	Sand clay

Abbreviation: MO, organic matter.

Since our study was conducted on‐farm in a perennial crop, and it was not possible to use plots or fields established specifically to attend all the needs of homogeneity between the treatments. In order to maximize homogeneity, we searched for areas containing both management systems in a paired fashion and presenting similar characteristics except for the management. Of all of the prospected sites in the region, only three attended to our standards; the details of soil characteristics, area, and slope are provided in Table 1. In this case, we opted to use a reduced number of areas, rather than to sample many areas that would add many confounding variables to our data set, following the scheme adopted by Cardoso et al. (2003). According to a meta‐analysis performed by Bengtsson et al. (2005), evaluating the effects of organic agriculture on species richness and abundance in on‐farm studies, prioritization either of the number of study sites or of homogeneity between the sites, can have consequences either way. In the first case, differences between treatments may be attributable to landscape differences, while in the second case, when matched site pairs are used, this can reduce the differences due to high similarities between the sites. To compensate for the reduced number of areas, we established a detailed sampling design of these three (Figure 1b) and opted for a local scale study.

### 2.2 Soil sampling

Soil samples were collected in 2010 from pairs of fields, such that each field of a pair was sampled on the same day, as follows: Area 1 (6th June): 97 agroforestry and 97 full‐sun samples; Area 2 (10th July): 78 agroforestry and 76 full‐sun samples; and Area 3 (17th September): 70 agroforestry and 72 full‐sun samples. This was during the dry season and included the coffee harvesting period. Samples were collected from points in a rough grid of nine or ten adjacent planted rows (*ca*. 3 m between each row) by eight to ten samples per row (every third bush, *ca*. 4 m long from each other). Sampling was done *ca*. 75 cm away from the coffee plant's trunk, just beneath the canopy, with the aid of a core soil sampler to 20 cm depth (Figure 1b). The core sampler was washed in water and then 70% ethanol between each sample. Soil samples were immediately transferred to individual polyethylene bags for transport to the laboratory.

A pooled soil sample of each field was sent for characterization of the soils' physical and chemical properties at the Laboratory of Soil Analysis in the Department of Soil Science at the Federal University of Viçosa, and the results are presented in Table 1. The surveyed soils were classified as clay soils except for the full‐sun field in Area 3 that was classified as sandy clay. The pH in all the fields ranged from 5.3 to 6.18, with most of the fields being classified as moderately acid (pH 5.6–6.0), except for the agroforestry field in Area 1 and the full‐sun field in Area 3, that were classified as slightly acid and strongly acid, respectively. All full‐sun fields presented lower pH values than their respective agroforestry field pair. The same pattern was observed for the organic matter values. Samples were individually mixed and homogenized manually. A subsample of soil was transferred from each of the plastic bags to a 200 ml transparent cup leaving some empty room at the top to keep the sample aired. Since the soils were collected in the dry season, samples in all the three areas were moistureless and 10 ml of sterile distilled water were added to each sample. In line with previous studies (Goble et al., 2010; Klingen et al., 2002; Meyling & Eilenberg, 2006), we did not use controls as it is nearly impossible to use a substrate that is similar to soil and which will not affect the bait insect's survival; even sterilized soil properties are totally modified by high temperatures (Ellis, 2004).

### 2.3 Insect bait method

As an indicator of potential ecosystem services, we used the insect bait method coupled with survival analyses and explored how long it takes for naturally occurring fungi in different soils to kill the bait insects. This method offers a simple measure of the activity of these entomopathogenic fungi in the soil and so helps to determine how crop management systems may favor or hinder this. Here, this adaptation of the insect bait method is referred to as the “bait‐survival technique” and the time to kill the baits as “speed of kill”. Larvae of the mealworm, *Tenebrio molitor* (Coleoptera: Tenebrionidae), were used as baits (Aguilera Sammaritano et al., 2016; Kim et al., 2018; Sanchez‐Pena, Lara, & Medina, 2011). This bait insect has been shown to be less susceptible to insect‐pathogenic fungi than the insect most commonly used as bait, *Galleria mellonella* (Lepidoptera: Pyralidae) (Bidochka, Menzies, & Kamp, 2002; Oreste, Bubici, Poliseno, Triggiani, & Tarasco, 2012), a characteristic we considered desirable here as it increases the relevance to soil‐dwelling insects. The insect larvae were obtained from a stock rearing maintained on wheat bran and chayote (a cucurbit). Larvae were *ca*. 2 months old and were of similar sizes (*ca*. 1.3 cm) when used–it is difficult under these circumstances to determine the exact instar (Morales‐Ramos, Rojas, Shapiro‐Ilan, & Tedders, 2010). Four larvae were added on to the soil surface in each cup (200 ml), and these were closed with perforated lids. Cups were shaken daily, inverted and left upside down (to force the insects to traverse the substrate) during the first week. Cups were then inspected every 3 days, and dead insects were surface‐sterilized with 70% ethanol, 5% sodium hypochlorite, two washes in sterile distilled water and dried on sterile filter paper.

All dead bait larvae presented signs of fungal infection and colonization after mortality, and no cadavers sporulated while within the soil pot. Hardened or mummified insect cadavers, with the body internally colonized by fungal hyphae, were the main symptom of death by entomopathogenic fungal infection. Cadavers were then transferred and incubated to moisture chambers (1.5‐µl sterile microtubes half‐filled with moistened cotton wool) to promote fungal growth.

### 2.4 Fungal isolation and identification

Dead insects were inspected under a stereomicroscope (40×) for external fungal growth and for preliminary fungal identification. All fungi detected were isolated on to plates with PDA (20% Potato, 2% Dextrose, and 1.5% Agar) and rifampicin and were incubated at 24°C in the dark. Fungi from these cultures were mounted on slides for microscopic observation (400×) and identified according to morphological characteristics. Isolates were preserved in silica gel and stored at 5°C.

### 2.5 Statistical procedures

A series of survival regression analyses was carried out to test the hypothesis of differential speed of kill of the bait insect between the two agroecosytems (Agroforestry vs. Full‐sun), with bait insect survival as the dependent factor and farming system (Agroforestry vs. Full‐sun) as an independent factor. For all three areas, survival data were analyzed for each pair of fields, considering each soil sample coming from the same field individually in the analysis. Although the survival data are clustered into groups of four individuals coming from each soil sample, we considered their survival independently in the survival analysis. To deal with this lack of independence in the groups of four insects in the same sample, we added the “frailty” function, from the R software package “frailtypack” (Rondeau et al., 2019), in the survival models. The function is a maximum penalized likelihood estimation that accounts for unobserved random proportionality factors coming from clustered individuals in survival analysis (Therneau, Grambsch, & Pankratz, 2003), adding random effects that act multiplicatively on the hazard function. In our analysis, a penalization was added to fit a joint frailty model of the four insects in same soil sample. The general procedure was as follows. Data from all three areas (i.e., pairs of fields) were analyzed with censored Weibull distributions and were compared by ANOVA using chi‐square tests (Crawley, 2007). The function “frailty” was added in the models with gamma distributions (Rondeau et al., 2012).

In the first analyses, all dead insects, whether or not they presented symptoms of fungal infection after death, were included in the survival analyses as it is common for fungi to infect and kill an insect yet not sporulate successfully from the cadavers (Elliot, Blanford, & Thomas, 2002, Garcia et al., 2016). This was done for three areas separately. Finally, we conducted a series of survival regression analyses considering separately which fungi were found to have emerged from the insect cadavers, as this may indicate causes of death. Thus, in one set of three analyses, we considered only insects from whose cadavers fungi emerged (*Fusarium*, *Beauveria*, *Metarhizium*, and *Isaria*). In a second set of analyses, we considered only the bait insects from which fungi of proven entomopathogenic capacity emerged. Although some *Fusarium* species do have proven entomopathogenicity (Sharma & Marques, 2018), here it was excluded from this set of analyses, since we did not complete Koch's Postulates or identify at species level isolates recovered here and some could be saprophytic or secondary infections. In a third set, we considered only the insects from which *Fusarium* alone emerged. In all cases, data from excluded causes were censored at the times recorded for death.

To examine the frequencies with which the fungi were found in bait insects, samples were scored as positive or negative for entomopathogenic fungi to compare totals between each pair of fields. Samples were considered positive if at least one bait insect was infected by *Beauveria*, *Metarhizium*, or *Isaria*. If more than one fungal genus was present in the same sample, they were considered together (*Metarhizium* spp. + *Beauveria* spp.). *Fusarium* was isolated from a considerable number of the dead bait insects and consequently was present in the most of the soil samples. The genus *Fusarium* can colonize the insect body secondarily after it is killed by an entomopathogen (Teetorbarsch & Roberts, 1983). Thus, as explained above it was not included in the frequency analysis when it emerged from cadavers together with a fungus of proven insect‐pathogenic ability. The independent variable was farming regime while the response variable was number of soil samples positive for at least one entomopathogenic fungus and analyses were conducted for each area (field pair) separately. For construction of the full generalized linear model (GLM), the dependent variable was given a quasipoisson distribution and was analyzed by ANOVA with chi‐square tests (Crawley, 2007). Throughout, we checked for data overdispersion and we conducted residual analyses to determine model acceptability and suitability of error distributions (Crawley, 2007). All analyses were performed in R software version 3.4.2 (R Development Core Team, 2017).

## 3 RESULTS

### 3.1 Fungal isolation from bait insects

After exposure to moist conditions, most of the cadavers presented growth of entomopathogenic fungi over the body surface. In some cases, insects that presented signs of death by entomopathogenic fungi did not present external fungal growth or presented only *Fusarium* growth. In some cadavers, it was possible to isolate the entomopathogenic fungi growing internally in the insect body; however, in other cases *Fusarium* overgrew the other fungi. Very few cadavers presented signs of infection by bacteria, and none were infected by entomopathogenic nematodes.

### 3.2 Speed of kill of the bait insects

**Figure 2:**
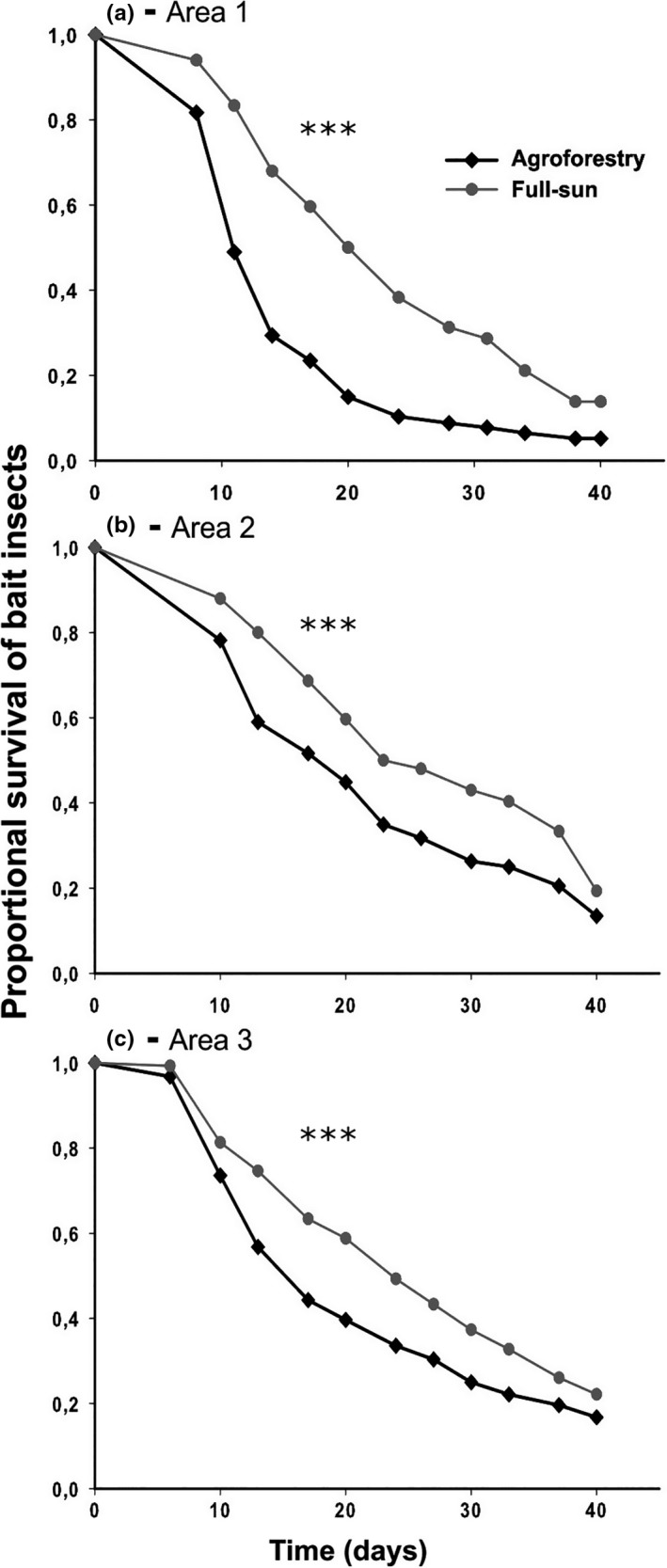
Differential survival of *Tenebrio molitor* (Coleoptera) bait insect larvae in soils from Agroforestry versus Full‐sun coffee farming systems in Minas Gerais, southeastern Brazil. Soils from six areas were sampled and were taken in pairs, each pair containing one field of each management type (Agroforestry vs. Full‐sun). Mortality of bait insects was evaluated for 40 days. Shown are proportional insect survivals for (a) Area 1; (b) Area 2, and (c) Area 3. Survival analyses are presented in the text. ***p < .001

Bait insects exposed to agroforestry soils in the three surveyed areas died 4–9 days faster than insects exposed to full‐sun soils (median survival times or LT_50_'s ± *SE* were as follows: Agroforestry vs. Full‐sun, Area 1: 11 ± 0.23 vs. 20 ± 0.48 days, χ^2^_[89]_ = 415.06; *p* < .001; Area 2: 17 ± 0.62 vs. 23 ± 0.94 days, χ^2^_[65.2]_ = 239.65, *p* < .001; Area 3: 13 ± 0.38 vs. 20 ± 0.99 days, χ^2^_[62.5]_ = 279.79; *p* < .001; Figure 2).

When we considered solely the insect cadavers from which fungi emerged (*Fusarium*, *Beauveria*, *Metarhizium*, and *Isaria*), the differences between the management systems were maintained in all the three areas (median survival times or LT_50_'s ± *SE* were as follows: Agroforestry vs. Full‐sun, Area 1: 11 ± 0.27 vs. 20 ± 0.57 days, χ^2^_[83.4]_ = 351.74; *p* < .001; Area 2: 13 ± 0.31 vs. 20 ± 1.1 days, χ^2^_[63.3]_ = 216.8, *p* < .001; Area 3: 17 ± 0.75 vs. 20 ± 1.08 days, χ^2^_[60.7]_ = 266.04; *p* = .006; Figure S1A–C). In the analyses considering solely the insect cadavers from which fungi with proven entomopathogenic capacity emerged (*Beauveria*, *Metarhizium*, and *Isaria*), the differences were maintained for Areas 1 and 2 (Agroforestry vs. Full‐sun, Area 1: 14 ± 0.35 LT_50_ ± *SE* vs. 20 ± 0.86 days, χ^2^_[75.8]_ = 238.1; *p* < .001; Area 2: 13 ± 0.40 vs. 20 ± 1.6 days, χ^2^_[58.6]_ = 184.1, *p* < .001; Area 3: 13 ± 0.46 vs. 17 ± 1.00 days, χ^2^_[56.1]_ = 198.38; *p* = .5; Figure S1D–F). When only the cadavers from which *Fusarium* emerged were considered, the differences were maintained only in Area 1 and Area 3 (median survival times or LT_50_'s ± *SE* were as follows: Agroforestry vs. Full‐sun, Area 1: 11 ± 0.41 LT_50_ vs. 20 ± 0.77 days, χ^2^_[80.2]_ = 234.45; *p* < .001; Area 2: 17 ± 1.18 vs. 23 ± 1.52 days, χ^2^_[55.7]_ = 125.55, *p* = .5; Area 3: 17 ± 1.05 vs. 24 ± 1.48 days, χ^2^_[58.2]_ = 169.56; *p* = .002; Figure S1G–I).

### 3.3 Fungal frequencies

**Figure 3:**
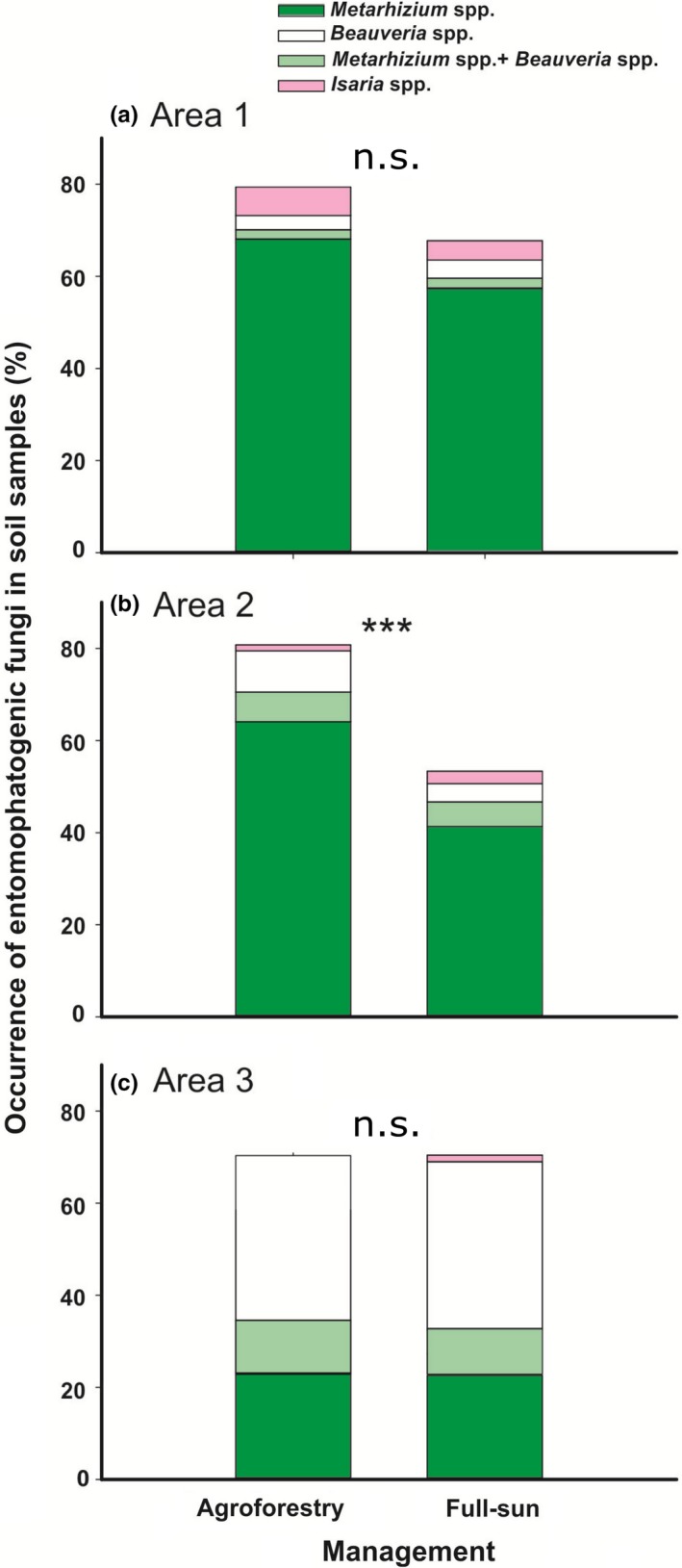
Mean (±*SE*) numbers of positive sample for insect‐pathogenic fungi from two coffee management systems: Agroforestry and full‐sun. Soil samples were taken from six paired coffee fields, that is, from three sites where samples could be taken from both systems: Area1 (a), Area 2 (b), and Area 3 (c) in Araponga, Minas Gerais, Southeastern Brazil. ***p < .001, n.s., not significant (see text)

Total frequencies of occurrence of entomopathogenic fungi (i.e., the number of samples that harbored at least one entomopathogenic fungus belonging to the genera *Beauveria*, *Isaria* or *Metarhizium*) were greater in Agroforestry soil than in full‐sun soil in Area 2 (Agroforestry vs. Full‐sun, Area: 0.81 ± 0.04 mean ± *SE* vs. 0.53 ± 0.06, χ^2^_[151]_ = 77.19, *p* < .001; Figure 3b). Frequencies in both fields of Area 1 and 3 were the same (Agroforestry vs. Full‐sun, Area 1: 0.79 ± 0.04 mean ± *SE* vs. 0.68 ± 0.04, χ^2^_[192]_ = 86.25; *p* = .06; Area 3: 0.70 ± 0.05 mean ± *SE* vs. 0.69 ± 0.05, χ^2^_[139]_ = 71.29; *p* = .89; Figure 3a,c).

## 4 DISCUSSION

We demonstrate here the idea that speed of kill of bait insects can be used to compare soils in terms of the danger they represent to insects. The different management systems—agroforestry and full‐sun—affect speed of kill of bait insects by entomopathogenic fungi. Previous studies have focused on abundance or species compositions (Garrido‐Jurado, Fernández‐Bravo, Campos, & Quesada‐Moraga, 2015; Goble et al., 2010; Klingen et al., 2002; Meyling & Eilenberg, 2006; Vanninen, 1996) rather than their activity. We propose that the speed of kill correlates positively with natural biological control of soil‐dwelling insect pests in the field, although we were not able to investigate this in the present study.

Our finding of greater activity of insect‐pathogenic fungi in organically managed agroforestry soils support the hypothesis that these systems have a positive effect on insect‐pathogenic fungi and could be more insect‐suppressive. Agroforestry management might contribute to the maintenance of the viability and virulence of these fungi and consequently confer insect pest‐suppressive potential to the soil. Litter produced by trees in agroforestries protects the soil against erosion, serves as food for soil organisms, and improves soil structure (Beedy, Snapp, Akinnifesi, & Sileshi, 2010). Shade cover also decreases solar radiation and increases microclimatic stability (Lin, 2007), which is particularly important as it is known that exposure to UV light is a major environmental factor affecting the action of those entomopathogens in the field (Braga, Flint, Miller, Anderson, & Roberts, 2001; Lovett & St. Leger, 2014). Shade and plant diversity in this system can also protect and promote functional biodiversity such as that of antagonists of pests and pathogens, and pollinators, reducing crop damage by herbivores, and increasing production (Iverson et al., 2014; Rezende et al., 2014; Tscharntke et al., 2011; Winqvist et al., 2011). Plant diversity also promotes more abundant and active populations of insect‐pathogenic fungi since these have been reported in mutualistic associations with plants acting as rhizosphere‐competent symbionts (Hu & St Leger, 2002) and endophytes (Barelli et al., 2018; Posada, Aime, Peterson, Rehner, & Vega, 2007).

Despite our evidence of insect‐suppressive potential in agroforestry soils, the cryptic nature of soil and the many interactions that take place in this environment could offer a range of alternative explanations to our results. One of these could be the presence of a more diverse microbial community in full‐sun soils that could competitively displace entomopathogenic fungi or delay their action in full‐sun soil samples. Given that we are looking for entomopathogenic activity, regardless of the mechanisms driven the entomopathogenic activity, the results are the faster mortality of insects in agroforestry than in full‐sun soils. Unraveling the mechanism behind a function or process and what are the taxonomic entities performing, it is clearly relevant; however, in many cases it is the ultimate service that is important. In a recent example, Wood et al. (2015) found that functional diversity is far more important as indicator of denitrification and carbon mineralization than abundance of genes and microbial taxonomic diversity when comparing tropical smallholder agroforestry systems and smallholder subject to mineral fertilization. This illustrates that the nature of future work on the service provided by soil entomopathogenic fungi may be far from obvious and liking entomopathogenic function to the number of fungal propagules per gram of soil can neglect important aspects.

Bait insect survival times can be used as an indicator of the insect‐suppressive potential provided by these fungi in agroecosystems. Generally, the methods employed to investigate naturally occurring entomopathogenic fungi in similar studies vary considerably, making comparison difficult. The method of isolation, whether bait method or cultivation in selective media, is the main source of divergence between studies (Hernández‐Domínguez, Cerroblanco‐Baxcajay, Alvarado‐Aragón, Hernández‐López, & Guzmán‐Franco, 2016; Medo & Cagáň, 2011). Here, we use a simple improvement of the insect bait method, including periodic evaluation of the bait mortality and analyzing this with survival analyses. In this manner, the differential mortality in different surveyed soils, visualized as differences in survival curves, will indicate the soil that offers greater insect‐suppressive potential. While abundance of fungal propagules in the soil could be considered an indicator of their function, this is only one component of their ecology, and their capacity to kill insects could be a far more relevant measure for inferences about their insect‐suppressive potential, since it captures more aspects of their activity.

Once an entomopathogenic fungus infects an insect, it takes time for infection development and host death (Hajek & St Leger, 1994). In experiments with the coffee berry borer, *Hypothenemus hampei* (Ferrari) (Coleoptera: Scolytidae), one of the most important pest insects of coffee crop cultivation, *Beauveria bassiana* and *Metarhizium anisopliae* took about 6–10 days to kill around 40%–90% of insects when inoculated directly at high concentrations (Neves & Hirose, 2005; Samuels, Pereira, & Gava, 2002). Here, 67.8% of the bait insect in agroforestry soils died after 20 days of exposure. If we consider that the concentration of fungal conidia in the soil was probably lower than in a laboratory assay, and the insects needed to move through the soil to enter in contact with them, the survival and time to death shown in agroforestry soils could be considered short.

Soil physical and chemical properties are known to influence the occurrence of entomopathogenic fungi. The proportions of silt and organic matter are known to correlate positively with their abundance (Medo & Cagáň, 2011; Quesada‐Moraga et al., 2007). The level of silt was very low in all the three sampled areas in our study, but the organic matter level was higher in all the agroforestry fields when compared in pairs with the respective full‐sun system. Quesada‐Moraga et al. (2007) reported that high clay content, pH, and low organic matter are positively correlated with *Beauveria* occurrence, and *Metarhizium* has a strong positive correlation with higher levels of organic matter. However, Medo and Cagáň (2011) reported divergent results, wherein higher occurrence of *Beauveria* was associated with high levels of organic matter, lower pH levels (5.5–6.5), and altitudes of ≤650 m. In our study, all the samples areas had pH of 5.3–6.2, organic matter of 2.9%–5.7%, clay contents of 39%–52%, and altitudes of 1,187 m. As our sampled areas were very close to one another, the soil proprieties did not show great variation and did not show any specific relation to the variation in the occurrence of the fungal genera.

As we used only morphological identification of the fungal isolates, it is likely that distinct species of the sampled genera were recovered in our study. While even closely related species can perform different functions, which could translate into differences in the ecosystems services they perform, what we propose here is a measure that is easy, cheap and can be applied more extensively and quickly than molecular identification or quantification of propagules. Naturally, species identities offer important information, but in many cases the “bait survival technique” can be a first step in the characterization of potential ecosystem services of a particular field or area, or a given management practice. Here, our goal was to investigate a possible ecosystem service, thus the function performed by these insect pathogens is far more important than fungal identity, much as one might expose prey insects in the field and see how many are removed by predators without needing to identify these. Nevertheless, the frequencies at genus level may offer some clues as to what is occurring in the field.

Regarding the fungal composition, Areas 1 and 2 were more related to each other than Area 3. *Metarhizium* was the dominant genus in Areas 1 and 2, and *Beauveria* was dominant in Area 3. This difference could be due to the differences in sample dates. The first two areas were sampled at the beginning of the coffee harvesting (June and July), and Area 3 was sampled at the end of the harvesting period (September), which could directly affect the fungal composition. *Beauveria bassiana* is frequently reported as a natural mortality factor in *H. hampei* (De La Rosa, Alatorre, Barrera, & Toriello, 2000). *Hypothemus hampei* infested berries fall to the soil (Damon, 2000) mainly by the end of the coffee harvesting. Insects infected with *B. bassiana* in the soil might act as inoculum source due to conidiation in cadavers (Bustillo, Bernal, Benavides, & Chaves, 1999). In other studies, *Beauveria* presence in the soil was also correlated with the presence of its insect host (Keller, Kessler, & Schweizer, 2003; Kessler, Enkerl, Schweize, & Keller, 2004).

The high levels of *Beauveria* spp. in Area 3 may also explain the low *Metarhizium* spp. levels in this area, as there is likely competition between these fungi for the bait insect. This is not to imply that *Metarhizium* spp. is at low levels in soil, but that it may be competitively excluded by *Beauveria* spp. within insect hosts in this particular period. *Metarhizium* spp. was the most frequently isolated of the fungi. This fungus has been reported to be more abundant in cultivated soils than in surrounding noncultivated soils from temperate regions (Bidochka et al., 1998; Jabbour & Barbercheck, 2009; Meyling & Eilenberg, 2006; Sanchez‐Pena et al., 2011; Sharma, Oliveira, Torres, et al., 2018; Sun & Liu, 2008; Vanninen, 1996). It is possible that this fungus is quite well‐adapted to survive in agricultural soils; however, even in agricultural soils there may be variability in the suitability of conditions. The use of *T. molitor* as bait could be another possible explanation for the frequency of *Metarhizium* spp., since Sharma, Oliveira, Torres, et al. (2018) report that this bait insect is biased to recover *Metarhizium robertsii* rather *B. bassiana*.

The genus *Fusarium* has previously been recovered with the insect bait method in soil samples in Palestine (Ali‐Shtayeh et al., 2003), Norway (Klingen et al., 2002), China (Sun & Liu, 2008), and Portugal (Sharma, Oliveira, Torres, et al., 2018). Although *Fusarium* species have been reported infecting several insect orders (Sharma & Marques, 2018; Teetorbarsch & Roberts, 1983), it was for long consider as a saprophyte rather than a true entomopathogen. However, a recent study reviewed the insecticidal activity of *Fusarium* species based on evidence from immunological, ecological, and experimental studies and concludes that some species are true entomopathogens (Sharma & Marques, 2018). Due to the versatile lifestyle of some species in the genus, it is also possible that they can switch between insect parasitism and other lifestyles, such as other animals and plant pathogens, endophytes, and saprophytes (Teetorbarsch & Roberts, 1983). In our study, *Fusarium* spp. was isolated at high frequencies and we speculate that this frequency could be due to primary and secondary infections following primary infections by an entomopathogenic fungus, since it was frequently isolated from the same bait insect also infected by other entomopathogens—*Beauveria* and *Metarhizium* are poor competitors for organic resources compared to opportunistic or saprophytic fungi that are ubiquitous in the soil (Goble et al., 2010). Also, *Fusarium oxysporum* has been reported to be resistant to many agricultural disturbances, being able to survive in many different soil conditions (Sharma, Oliveira, Raimundo, Torres, & Marques, 2018), which is in accordance with the frequency of *Fusarium* spp. observed in both management systems studied here.

## 5 CONCLUSION

Our study enabled us to detect apparently positive effects of an agroforestry management system on the activity of insect‐pathogenic fungi and their abundance, when compared with full‐sun systems. This finding suggests that agroforestry systems may improve the action of insect‐pathogenic fungi as has previously been shown for other functional groups such as hymenopteran parasitoids and for processes such as decomposition (Beedy et al., 2010, Leakey, 2014, Rezende et al., 2014, Winqvist et al., 2011). Our study has limitations and biases such as the limited sample size and geographical range, the use of just one bait, or the limited number of baits per samples; despite that, our finding is novel for insect‐pathogenic fungi. However, we suggest that we have taken the first steps to reveal and explore a very important ecosystem service, one requiring, ultimately, conservation in order to exploit it to the fullest. Further research can determine if our finding is a general pattern with the application of the bait survival technique in surveys. A number of other questions arise from this study. Among the most relevant are whether environmental diversity is indeed responsible for the high mortality rates in agroforestry and what the relationship is between environmental diversity and fungal genetic diversity. We still have only limited information about how environmental factors affect the abundance and activity of insect‐pathogenic Hypocreales but we suspect that they may often maintain a symbiotic relationship with plants such as coffee, or with the other co‐occurring plants, and this may in part contribute to the success of agroforestry systems.

## CONFLICT OF INTEREST

None declared.

## AUTHOR CONTRIBUTIONS

CCM, IMC, and SLE conceived and designed the experiments. CCM and DC performed the experiments. CCM, TGS, and SLE analyzed the data. CCM and SLE wrote the paper. IMC and TGS revised the manuscript. CCM, TGS, and SLE originally formulated the idea. All the authors have approved the final version of the manuscript.

## Data Availability

Data supporting the results presented here are available at Dryad Digital Repository (https://doi.org/10.5061/dryad.t91r232).
