# Supplementary material for: Agroforestry coffee soils increase the insect‐suppressive potential offered by entomopathogenic fungi over full‐sun soils: A case proposing a “bait survival technique”
Source: Ecol Evol. 2019 Aug 30;9(18):10777–87. doi: 10.1002/ece3.5598 (PMC6787780; doi:10.1002/ece3.5598)
Supplement: Supplementary file 1 [file ECE3-9-10777-s001.docx]

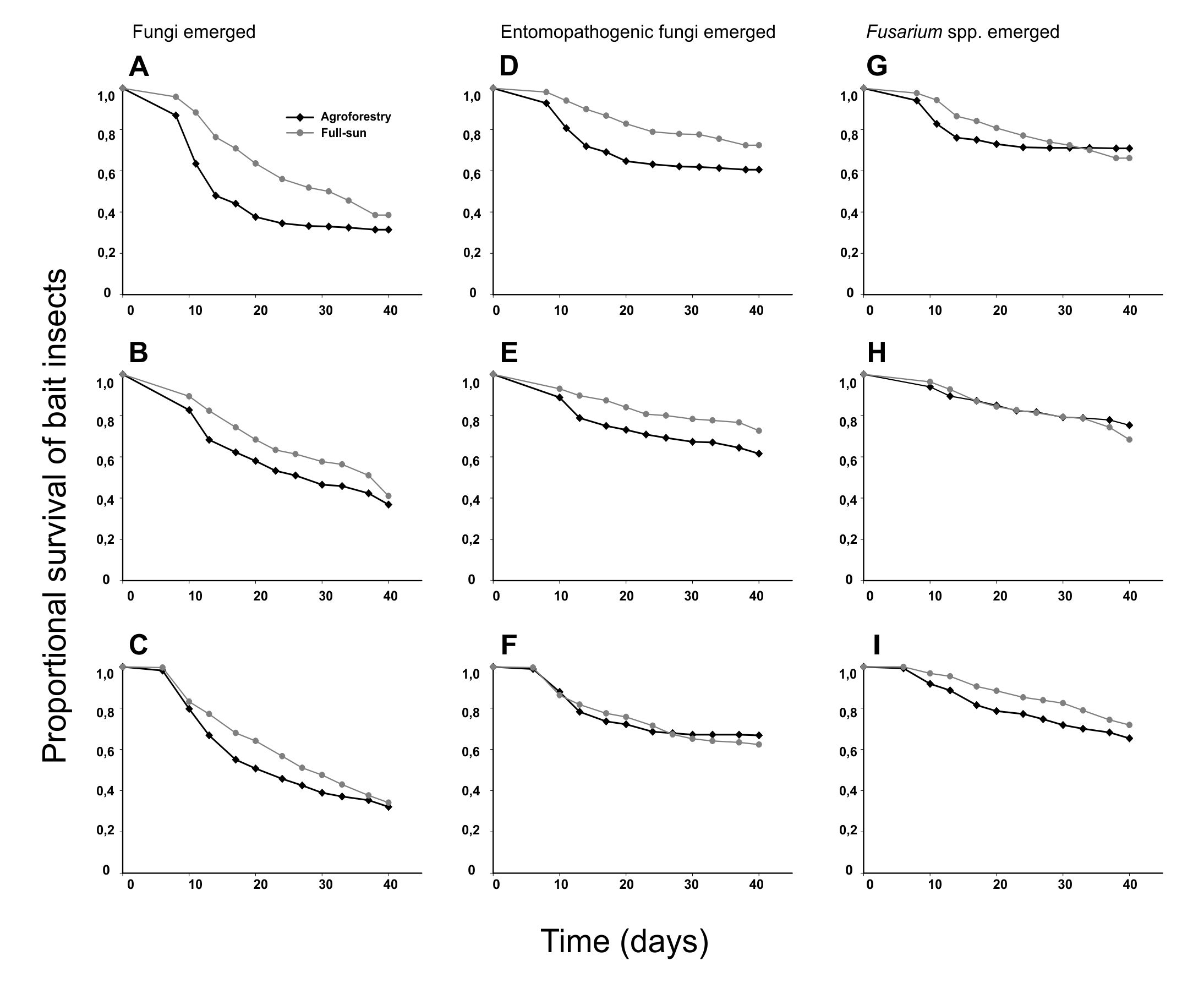


Fig. S1. Survival of bait insects (*Tenebrio molitor*; Coleoptera) exposed to soils from Agroforestry and Conventional coffee production systems in Araponga, Minas Gerais, Brazil. Shown are survival data according to which fungi emerged from cadavers. First column (A – C): Bait insects from which fungi emerged (*Beauveria* spp.*, Metarhizium* spp.*, Isaria* spp. and *Fusarium* spp.). Second column (D – F): Bait insects from which entomopathogenic fungi emerged (*Beauveria* spp.*, Metarhizium* spp. and *Isaria* spp.). Third column (G – I): Bait insects from which *Fusarium* spp. emerged. Area 1 is shown on the first row, Area 2 on the second and Area 3 on the third.
