# Supplementary material for: Agroforestry coffee soils increase the insect‐suppressive potential offered by entomopathogenic fungi over full‐sun soils: A case proposing a “bait survival technique”
Source: Ecol Evol. 2019 Aug 30;9(18):10777–87. doi: 10.1002/ece3.5598 (PMC6787780; doi:10.1002/ece3.5598)
Supplement: Supplementary file 2 [file ECE3-9-10777-s002.pdf]

Supplementary Data

R Script for the survival and frequency analysis

Survival analyses

#Time: days until death of the bait-insect

#management: agroforestry or full-sun

#sample: soil sample containing four baits insects

#Packages

library(survival)

library(frailtypack)

#Reading original dataset

data.area1 <- read.table("surv.area1.txt",h=T)

#attaching data

attach(data.area1)

#The bait-insects were censored in accordance with their death cause to check the influence of the different causes at the mortality.

#The function survreg were used with weibull distribution to analyse the diferential survival between both 'MANAGEMENT' surveyed. The function 'fraity.gamma' were used to penalize for the lack of independence of the bait-insects that came from the same soil sample.

In the first model, 'TOTAL' includes all dead bait-insects regardless of the cause of the death.

m1 <- survreg(Surv(Time,Total==1)~Management+frailty.gamma(Sample), data=data.area1)

anova(m1,test="Chi")

summary(m1)

In the second model, only the bait-insects were fungi from the genera Beauveria, Isaria and Metarhizium, 'EPF', were included and the bait-insect killed by other fungi or of unknow causes were censored.

m2 <- survreg(Surv(Time, EPF==1)~Management+frailty.gamma(Sample), data=data.area3)

anova(m2,test="Chi")

summary(m2)

#The third model includes the insects included in the second model plus the bait-insects were Fusarium was isolated.

m3 <- survreg(Surv(Time, EPFFus==1)~Management + frailty.gamma(Sample), data=data.area3)

anova(m3,test="Chi")

summary(m3)

The forth model includes only the bait-insects were Metarhizium were isolated 'Met'

m4 <- survreg(Surv(Time, Met==1)~Management+frailty.gamma(Sample), data=data.area3)

anova(m4,test="Chi")

summary(m4)

The last model includes only the baits were Fusarium were isolated 'Fusarium'

m5 <- survreg(Surv(Time, Fusarium==1)~Management+frailty.gamma(Sample), data=data.area3)

anova(m5,test="Chi")

summary(m5)

#Calculatiing the speed of kill (days until death of the insect-bait) and standard error for each of the death causes in the above models

tapply(Time[Total==1],Management[Total==1],median)

tapply(Time[Total==1],Management[Total==1],var)

sd.med<-tapply(Time[Total==1],Management[Total==1],mad)

sd.med

numobs<-tapply(Time[Total==1],Management[Total==1],length)

se<-sd.med/sqrt(numobs)

se

tapply(Time[EPFFus==1],Management[EPFFus==1],median)

tapply(Time[EPFFus==1],Management[EPFFus==1],var)

sd.med2<-tapply(Time[EPFFus==1],Management[EPFFus==1],mad)

sd.med2

numobs<-tapply(Time[EPFFus==1],Management[EPFFus==1],length)

se2<-sd.med2/sqrt(numobs)

se2

```r
tapply(Time[EPF==1],Management[EPF==1],median)
sd.med3<-tapply(Time[EPF==1],Management[EPF==1],mad)
sd.med3
numobs<-tapply(Time[EPF==1],Management[EPF==1],length)
se3<-sd.med3/sqrt(numobs)
se3

tapply(Time[Fusarium==1],Management[Fusarium==1],median)
sd.med4<-tapply(Time[Fusarium==1],Management[Fusarium==1],mad)
sd.med4
numobs<-tapply(Time[Fusarium==1],Management[Fusarium==1],length)
se4<-sd.med4/sqrt(numobs)
se4

#Frequency Analyses
#The analyses were conducted for each area (field pair) separately.
#fungal.frequency: number of soil samples that were recorded as positive for entomopathogenic fungi.
#samples were considered positive if at one bait-insect of the sample presented signs of infection by an entomopathogenic fungi
#management: Agroforestry and full-sun
#Reading original data
data.frequency <- read.table("frequency_area1.txt",header=T)
#attaching data
attach(data.frequency)
#checking data
summary(data.frequency)

#Generalized linear model constructed with the independent variable, management, and the response variable, number of soil samples positive for at least one entomopathogenic fungus with quasipoisson distribution
m6<-glm(fungal.frequency~management,quasipoisson)
```

```
anova(m6,test="Chi")

summary(m6)

#checking data overdispersion

plot(m6,which=1)

#calculating paramenters

mean.frequency <- tapply(fungal.frequency,management,mean)

mean.frequency

sd.error<-tapply
(fungal.frequency,list(management),sd)/sqrt(tapply(fungal.frequency,list(management),l
ength))

sd.error
```
